# Mild Effects of Sunscreen Agents on a Marine Flatfish: Oxidative Stress, Energetic Profiles, Neurotoxicity and Behaviour in Response to Titanium Dioxide Nanoparticles and Oxybenzone

**DOI:** 10.3390/ijms22041567

**Published:** 2021-02-04

**Authors:** Ana Carvalhais, Bárbara Pereira, Mariangela Sabato, Rafaela Seixas, Marina Dolbeth, Ana Marques, Sofia Guilherme, Patrícia Pereira, Mário Pacheco, Cláudia Mieiro

**Affiliations:** 1 CESAM and Department of Biology, University of Aveiro, 3810-193 Aveiro, Portugal; ana.carvalhais@ua.pt (A.C.); blpereira@ua.pt (B.P.); rafaelaseixas@ua.pt (R.S.); anammarques@ua.pt (A.M.); sofia.g.guilherme@ua.pt (S.G.); pkpereira@ua.pt (P.P.); mpacheco@ua.pt (M.P.); 2 Department of Biological and Environmental Sciences, Università degli Studi di Messina, 98166 Messina, Italy; sabmaryange@virgilio.it; 3 CIIMAR, University of Porto, 4450-208 Matosinhos, Portugal; mdolbeth@ciimar.up.pt or

**Keywords:** UV filters, mixtures, marine fish, environmental concentrations, oxidative stress, bioenergetics, neurotoxicity, behaviour

## Abstract

UV filters are potentially harmful to marine organisms. Given their worldwide dissemination and the scarcity of studies on marine fish, we evaluated the toxicity of an organic (oxybenzone) and an inorganic (titanium dioxide nanoparticles) UV filter, individually and in a binary mixture, in the turbot (*Scophthalmus maximus*). Fish were intraperitoneally injected and a multi-level assessment was carried out 3 and 7 days later. Oxybenzone and titanium dioxide nanoparticles induced mild effects on turbot, both isolated and in mixture. Neither oxidative stress (intestine, liver and kidney) nor neurotoxicity (brain) was found. However, liver metabolic function was altered after 7 days, suggesting the impairment of the aerobic metabolism. An increased motility rate in oxybenzone treatment was the only behavioural alteration (day 7). The intestine and liver were preferentially targeted, while kidney and brain were unaffected. Both infra- and supra-additive interactions were perceived, with a toxicodynamic nature, resulting either in favourable or unfavourable toxicological outcomes, which were markedly dependent on the organ, parameter and post-injection time. The combined exposure to the UV filters did not show a consistent increment in toxicity in comparison with the isolated exposures, which is an ecologically relevant finding providing key information towards the formulation of environmentally safe sunscreen products.

## 1. Introduction

The main role of sunscreens is to protect the skin against the harmful effects of ultraviolet (UV) radiation and their use is highly recommended to prevent sunburn and skin cancer [1]. The awareness of sunburn risk increased the consumption of cosmetics and sunscreens, particularly in coastal areas, promoting their spread into the aquatic environment [2]. These products and their components (e.g., UV filters and fragrances) are released into the water during bathing and washing activities [3] and their presence in marine systems has already been detected worldwide in concentrations ranging from 6.9 to 37.6 mg L^−1^ for titanium dioxide nanoparticles (TiO_2_ NP) and from 25.3 to 1223.2 ng L^−1^ for oxybenzone (BP-3) [4,5,6,7]. Despite this, the knowledge regarding their effects is still limited, and they are therefore considered as emerging contaminants [2,5].

UV filters are a major component of sunscreens, which can be organic (chemical) or inorganic (physical) filters. Organic filters have, as a mechanism of action, the absorption of UV rays and their conversion into thermal energy at a specific wavelength [8]. Aromatic compounds, such benzophenones (e.g., oxybenzone (BP-3) and avobenzone), camphor (4-methylbenzylidene camphor) and salicylates (e.g., 2-ethylhexyl salicylate and isoamyl 4-methoxycinnamate), are recurrently used for this purpose [3], and most of them have a specific wavelength spectrum of action: UVA (320–400 nm) or UVB (290–320 nm). Inorganic filters have a larger range of protection, being able to cover the entire UV spectrum and act as a physical barrier by reflecting and scattering the sunlight. Titanium dioxide (TiO_2_) and zinc oxide (ZnO) are the two most common physical UV filters used, working as a shield to the UV rays [9,10]. Nowadays, these two compounds usually appear in nanoparticle (NP) sizes to improve the texture and spreadability of the sunscreen, making these products more appealing cosmetically [11,12]. Moreover, the combination of both types of UV filters (organic and inorganic) is more effective and more frequently used in sunscreen composition, since it increases the protection factor [13,14].

Several studies demonstrated that these compounds have the ability to bioaccumulate and biomagnify in the trophic chains of the aquatic ecosystems, with potential harmful effects for both the environment and wildlife [2,10,13]. Organic UV filters, such as camphor, salicylates and BP-3, are highly lipophilic [15], being rapidly bioaccumulated in aquatic organisms, such as corals, crustaceans, mussels and fish [1,16,17,18,19,20,21,22,23,24]. BP-3 is partially metabolized and then excreted or accumulated in different fish tissues. For instance, three metabolites—4-dihydroxybenzophenone, 4-hydroxybenzophenone and 4,4′-dihydroxybenzophenone—were already found in the liver, gills and muscle of a marine fish [22]. The bioaccumulation of inorganic UV filters, such as TiO_2_ NPs, was also reported in several tissues of bivalves (e.g., digestive gland, gills and whole tissue) and fish (e.g., kidney, liver, gills, heart, brain and muscle) [25,26,27,28].

UV filters may cause toxic effects on several organisms such as humans, rats and aquatic organisms [16,18,29,30]. However, the existing data are not consistent, particularly regarding BP-3 and TiO_2_ NPs toxicity. The majority of the information regarding the impact of BP-3 in the marine environment is related to its effects on corals, reporting reef bleaching and mortality through oxidative-stress-mediated processes [31]. However, there is not much information for other marine invertebrates and for vertebrates, with the majority of studies reporting effects on freshwater fish species. Data from fish revealed contradictory results, depending on the tested concentrations; while some authors demonstrated impairment of both endocrine and reproductive functions [16], others stated that the endocrine disruptive ability of BP-3 is negligible at environmental levels [32]. Moreover, Rodríguez-Fuentes et al. [33] reported no oxidative stress in *Danio rerio*. Similarly to BP-3, the available literature on the potential toxic effects of TiO_2_ NPs in marine organisms points to a wide range of sometimes contrasting effects: moderate histological effects [34], changes in immunological parameters [35,36], oxidative stress [35,37,38], absence of DNA damage [39] and cyto- and genotoxicity [26].

Bearing in mind the controversy of results regarding BP-3 and TiO_2_ NPs, it is crucial that the set of endpoints selected to evaluate their toxicity to fish is highly sensitive and able to detect sub-lethal effects in a high number of species. Biomarkers of effects, such as oxidative stress parameters, metabolic profile and neurotoxicity, are recognized as suitable tools for environmental risk assessment in fish [40,41], providing rapid responses at the sub-organismal level that translate relevant adverse molecular processes [40]. Even if those parameters translate subtle effects, they should not be neglected as they can anticipate long-term adverse effects and predict the health status of the organism [42]. Behaviour-based biomarkers are also acknowledged as valuable to address the effects of aquatic contaminants in fish by providing an integrated whole-organism response [43,44]. Indeed, changes in fish behaviour are an apical manifestation of alterations directly on the central and peripheral nervous systems or indirectly through the activation of physiological functions, such as metabolism [43]. Since fish behaviour assessment provides an irreplaceable perspective for the evaluation of aquatic contaminants’ toxicity by linking effects on physiology to ecological repercussions, there has been increasing interest in including behavioural endpoints in studies performed on this scope. Interestingly, research on the effects of TiO_2_ NPs and BP-3 has not yet followed the current tendency to embrace behavioural assessment in toxicological investigative studies. In fact, there are only a limited number of publications on the effects of these emerging contaminants in fish behaviour. The available studies focusing on the effects of BP-3 and TiO_2_ NPs, despite reinforcing the relevancy of behavioural analysis in the toxicological context, do not demonstrated a clear pattern of responses. While BP-3 showed that the type and severity of the behavioural effects depend on the exposure via [24,45] and the tested concentrations, TiO_2_ NPs were demonstrated to induce subtle alterations in fish swimming behaviour [46].

Oxidative stress, metabolic, neurotoxicity and behaviour biomarkers are not specific to a class of contaminants being suitable to screen the potential effects of several xenobiotics, isolated or in mixture. In fact, these parameters are already been described to assess both BP-3 and TiO_2_ NPs toxicity [24,28,33,37,38,45,47,48].

Considering the widespread use of TiO_2_ NPs and BP-3 and its occurrence in coastal waters, including in concomitance, it is crucial to increase the body of knowledge on the potential toxic effects on marine fish, related to environmental ranges of occurrence. Therefore, the main objective of this study was to evaluate the toxicity of isolated and binary mixtures of an organic (BP-3) and an inorganic (TiO_2_ NPs) UV filter to a marine flatfish (*Scophthalmus maximus*), using a multi-level approach, including oxidative stress, metabolic profile, neurotoxicity and behaviour.

## 2. Results

### 2.1. TiO_2_ Nanoparticles Characterization

**Figure 1:**
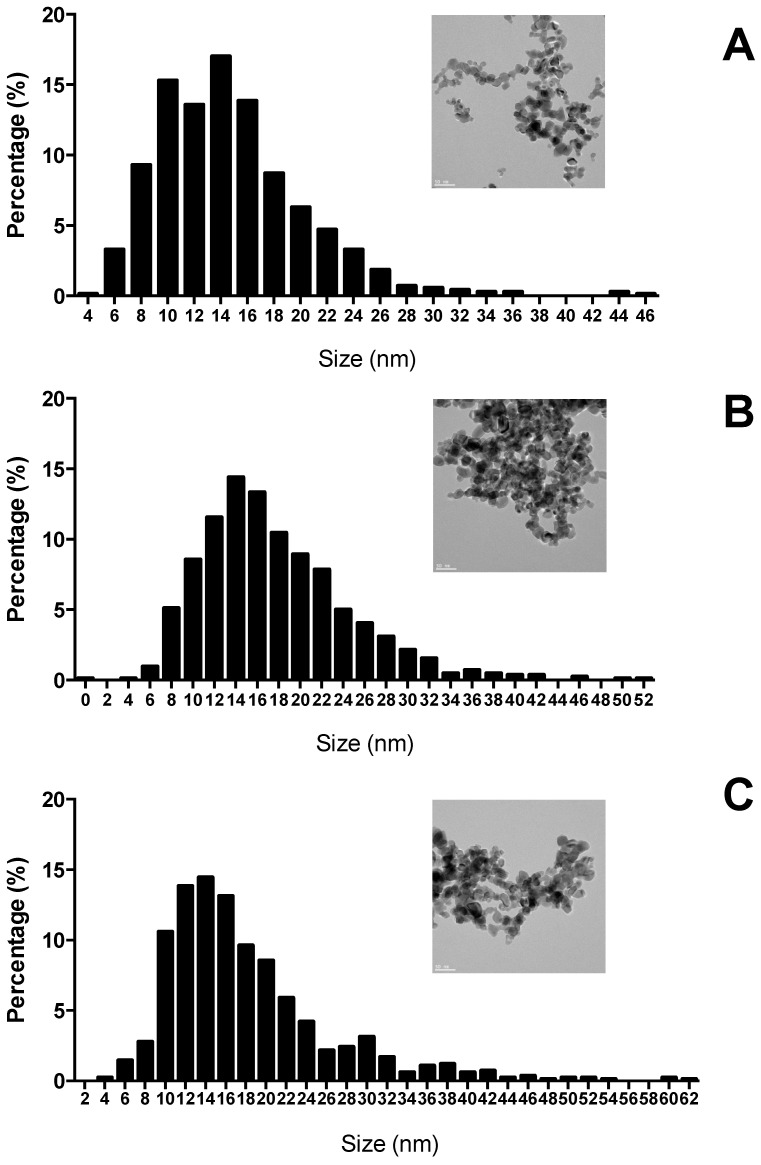
Scanning transmission electron microscope (STEM) images of titanium dioxide nanoparticles (TiO_2_ NPs) suspensions (scale 50 nm) and their size distribution histogram. (**A**) stock suspension in distilled water; (**B**) exposure suspension in saline solution (NaCl 9 g L^–1^) and DMSO; (**C**) mixture of TiO_2_ NPs + oxybenzone (BP-3) exposure suspension in NaCl (9 g L^–1^) and DMSO.

The XRD analysis demonstrated the presence of the crystalline phase anatase (86.8%) and rutile (13.2%). The standard spheroid irregular shape of TiO_2_ NPs (Aeroxide© P25) with a primary size of a mean particle diameter of 14.75 ± 5.62 nm in the stock solution is confirmed by STEM (Figure 1). The histogram of different sizes of TiO_2_ NPs showed a unimodal right skewed distribution, with 60% of the values ranging from 10 to 16 nm in the stock solution, 50% ranging from 12 to 18 nm in TiO_2_ NPs solution and 51% from 10–18 nm in the mixture.

### 2.2. Organ-Specific Oxidative Stress Profiles

#### 2.2.1. Responses in the Intestine

**Figure 2:**
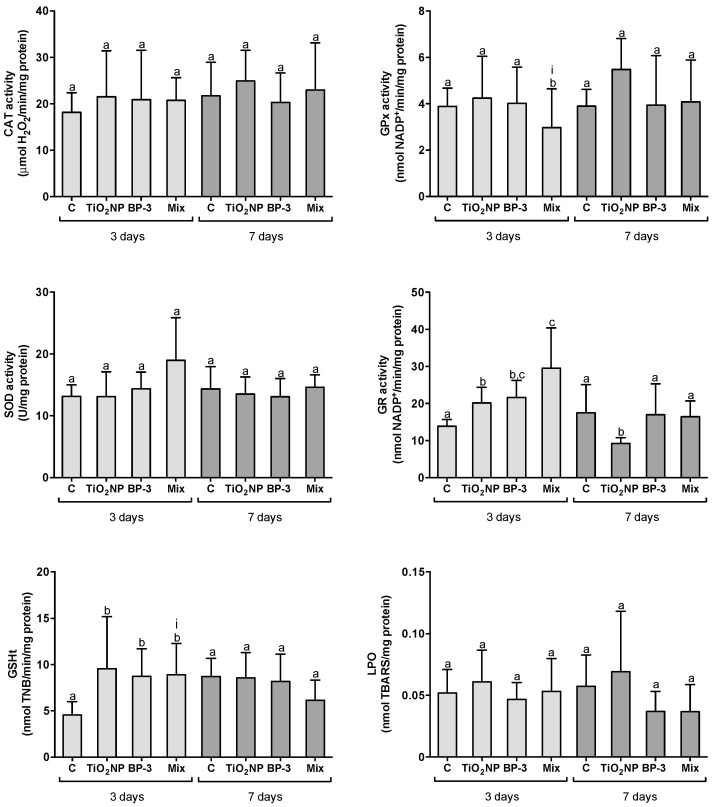
Oxidative stress responses in the intestine of *S. maximus*, 3 and 7 days after intraperitoneal injection of titanium dioxide nanoparticles (TiO_2_ NPs; 3.0 μg TiO_2_ NP per g fish weight), oxybenzone (BP-3; 3.0 μg BP-3 per g fish weight) or their mixture (Mix), as well as the respective control group (C). Different lower-case letters denote significant differences (*p* < 0.05). Significant interaction between TiO_2_ NPs and BP-3 is depicted by (i) (*p* < 0.05). Columns correspond to mean and error bars represent the standard deviation.

After 3 days of IP injection of TiO_2_ NPs, BP-3 and their mixture, the activity of CAT and SOD in the intestine was similar in all treatments (F = 0.9350, *p* = 0.4352; F = 2.2581, *p* = 0.1006 for CAT and SOD, respectively). However, the activity of GR and GSHt content increased in all treatments regarding the control (F = 16.2, *p* < 0.0001; F = 8.18603, *p* < 0.0001 for GR and GSHt, respectively). GR activity was also higher in the mixture in comparison with TiO_2_ NPs. In opposition to GR, the activity of GPx decreased in the mixture in comparison with all the other treatments (F = 7.4068, *p* < 0.0001). The interaction between TiO_2_ NPs and BP-3 was significant for GPx (L-ratio = 4.8288, *p* = 0.028) and GSHt content (F = 3.9146, *p* = 0.047). Despite the observed alterations on the antioxidant responses, no LPO was observed in the isolated UV filters or in the mixture (F = 0.6448, *p* = 0.592) (Figure 2). After 7 days, the activities of CAT (F = 0.81, *p* = 0.499), GPx (F = 1.983, *p* = 0.140) and SOD (F = 0.506, *p* = 0.681), as well as GSHt content (F = 2.001, *p* = 0.137), were similar among all treatments. The activity of GR decreased in TiO_2_ NPs in comparison to all the other treatments (F = 10.714, *p* < 0.001). No LPO alterations were found (F = 2.3117, *p* = 0.098) (Figure 2).

#### 2.2.2. Responses in the Kidney

**Figure 3:**
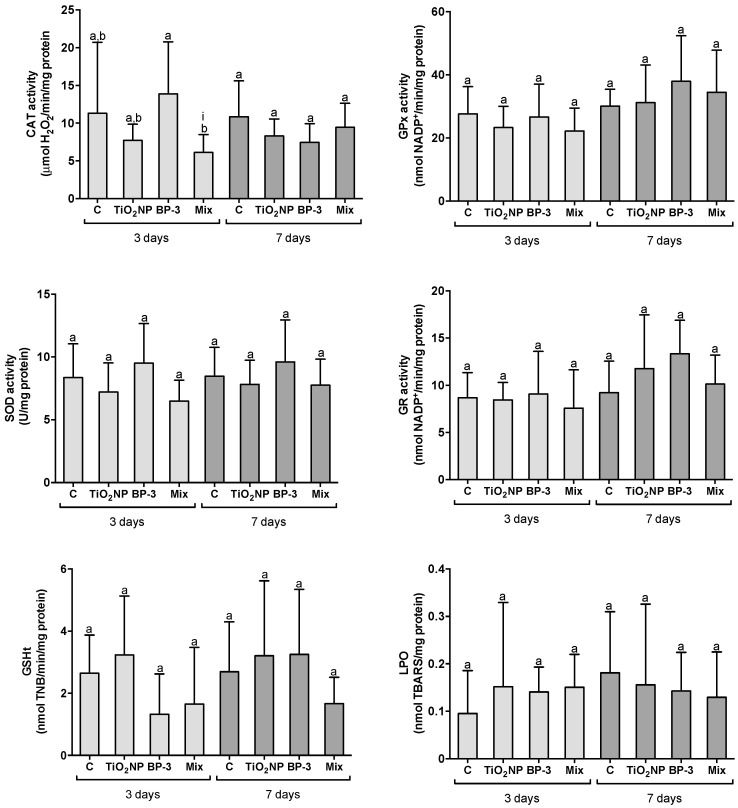
Oxidative stress responses in the kidney of *S. maximus*, 3 and 7 days after intraperitoneal injection of titanium dioxide nanoparticles (TiO_2_ NPs; 3.0 μg TiO_2_ NP per g fish weight), oxybenzone (BP-3; 3.0 μg BP-3 per g fish weight) or their mixture (Mix), as well as the respective control group (C). Different lower-case letters denote significant differences (*p* < 0.05). Significant interaction between TiO_2_ NPs and BP-3 is depicted by (i) (*p* < 0.05). Columns correspond to mean and error bars represent the standard deviation.

After 3 days, the activity of CAT was significantly lower in the mixture in comparison with BP-3 (F = 3.7052, *p* = 0.024), while all the other antioxidants and LPO remain similar in all conditions (GPx: F = 0.7188, *p* = 0.549; SOD: F = 0.931, *p* = 0.441; GR: F = 0.2225, *p* = 0.879; GSHt: F = 1.752, *p* = 0.187; LPO: F = 1.4417, *p* = 0.250) (Figure 3). The interaction between TiO_2_ NPs and BP-3 was significant for CAT (L-ratio = 4.2751, *p* = 0.048). After 7 days, all the antioxidants and LPO remained unaltered among treatments (CAT: F = 3.7444, *p* = 0.291; GPx: F = 0.8714, *p* = 0.470; SOD: F = 0.931, *p* = 0.441; GR: F = 1.6967, *p* = 0.196; GSHt: F = 1.1335, *p* = 0.360, LPO: F = 0.3253, *p* = 0.807) (Figure 3).

#### 2.2.3. Responses in the Liver

**Figure 4:**
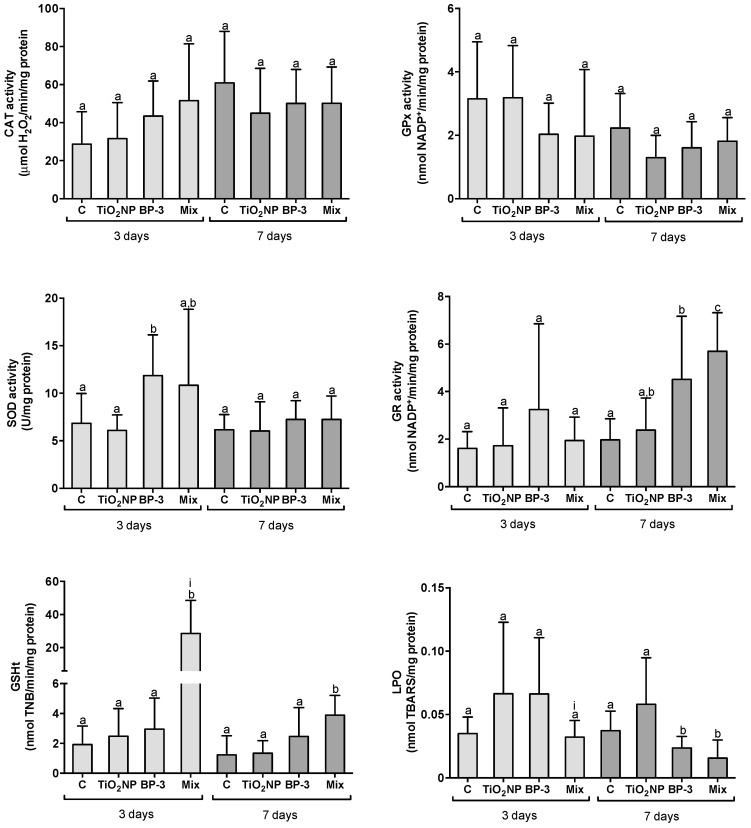
Oxidative stress responses in the liver of *S. maximus*, 3 and 7 days after intraperitoneal injection of titanium dioxide nanoparticles (TiO_2_ NPs; 3.0 μg TiO_2_ NP per g fish weight), oxybenzone (BP-3; 3.0 μg BP-3 per g fish weight) or their mixture (Mix), as well as the respective control group (C). Different lower-case letters denote significant differences (*p* < 0.05). Significant interaction between TiO_2_ NPs and BP-3 is depicted by (i) (*p* < 0.05). Columns correspond to mean and error bars represent the standard deviation.

Three days after IP injection, the activity of SOD significantly increased in BP-3 treatment in comparison to control and TiO_2_ NPs (F = 5.7379, *p* = 0.003), and the GSHt content increased in the mixture relatively to all the other treatments (F = 14.8997, *p* < 0.001). The remaining antioxidants and LPO were similar among treatments (CAT: F = 2.1913, *p* = 0.108; GPx: F = 1.3785, *p* = 0.268, GR: F = 0.3912, *p* = 0.760, LPO: F = 1.82541, *p* = 0.164) (Figure 4). The interaction between TiO_2_ NPs and BP-3 was significant for GSHt (L-ratio = 14.8204, *p* < 0.001) and for LPO (L-ratio = 5.070, *p* = 0.02). After 7 days, the activity of GR increased in BP-3 and in mixture in comparison to control, as well as in the mixture relative to TiO_2_ NPs (F = 13.6077, *p* < 0.0001). The content of GSHt significantly increased in the mixture in relation to all the treatments (F = 8.2677, *p* = 0.0007). LPO significantly decreased in BP-3 and in the mixture comparatively to control and to TiO_2_ NPs (F = 4.4169, *p* = 0.01). CAT, GPx and SOD activities were similar among treatments (CAT: F = 0.7003, *p* = 0.560; GPx: F = 1.416, *p* = 0.258 and SOD: F = 0.692, *p* = 0.565) (Figure 4).

### 2.3. Hepatic Metabolic Profile

**Figure 5:**
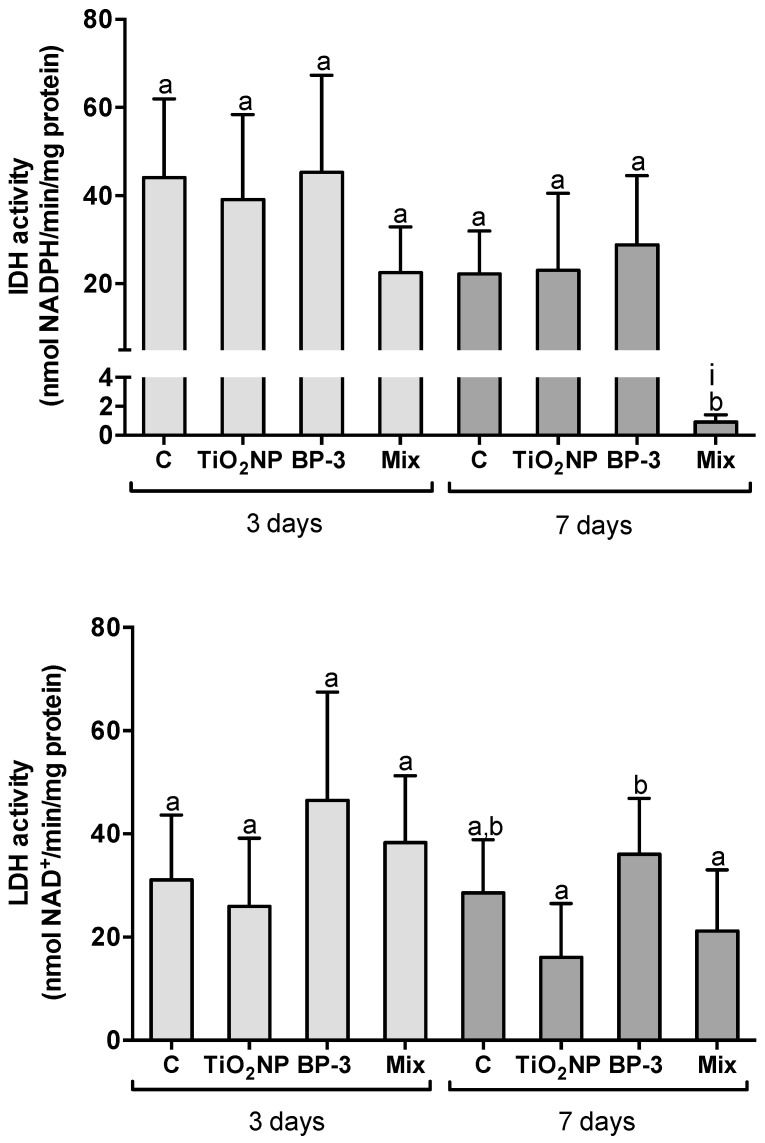
Metabolic profile of the liver of *S. maximus* assessed by isocitrate dehydrogenase (IDH) and lactate dehydrogenase (LDH), 3 and 7 days after intraperitoneal injection of titanium dioxide nanoparticles (TiO_2_ NPs; 3.0 μg TiO_2_ NP per g fish weight), oxybenzone (BP-3; 3.0 μg BP-3 per g fish weight) or their mixture (Mix), as well as the respective control group (C). Different lower-case letters denote significant differences (*p* < 0.05). Significant interaction between TiO_2_ NPs and BP-3 is depicted by (i) (*p* < 0.05). Columns correspond to mean and error bars represent the standard deviation.

IDH and LDH activities were similar among treatments after 3 days of IP injection (F = 2.7691, *p* = 0.06 and F = 2.8247, *p* = 0.05 for IDH and LDH, respectively), while after 7 days IDH decreased in the mixture relative to the other treatments (F = 26.456, *p* < 0.001). LDH increased in BP-3 treatment relative to TiO_2_ NPs and the mixture (F = 4.6682, *p* = 0.009) (Figure 5). After 7 days, the interaction between TiO_2_ NPs and BP-3 was significant for IDH (L-ratio = 6.7348, *p* = 0.009) (Figure 5).

### 2.4. Neurotoxicity

**Figure 6:**
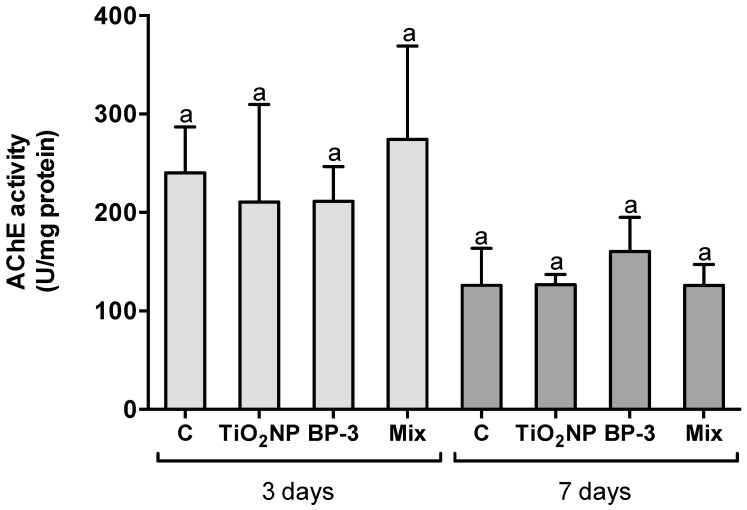
Neurotoxic profile depicted in the brain acetylcholinesterase (AChE) activity of *S. maximus*, 3 and 7 days after intraperitoneal injection of titanium dioxide nanoparticles (TiO_2_ NPs; 3.0 μg TiO_2_ NP per g fish weight), oxybenzone (BP-3; 3.0 μg BP-3 per g fish weight) or their mixture (Mix), as well as the respective control group (C). Different lower-case letters denote significant differences (*p* < 0.05). Columns correspond to mean and error bars represent the standard deviation.

No alteration in the AChE activity was observed in the brain after 3 (F = 1.6620, *p* = 0.197) and 7 days (F = 2.0198, *p* = 0.151) for TiO_2_ NPs, BP-3 and the mixture (Figure 6).

### 2.5. Behavioural Alterations

**Figure 7:**
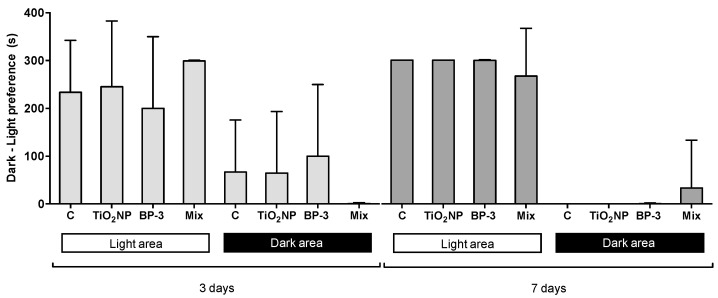
Dark-light preference analysis [seconds (s) spent in each area] of *S. maximus*, 3 and 7 days after intraperitoneal injection of titanium dioxide nanoparticles (TiO_2_ NPs; 3.0 μg TiO_2_ NP per g fish weight), oxybenzone [(BP-3; 3.0 μg BP-3 per g fish weight) or their mixture (Mix)], as well as the respective control group (C). Columns correspond to mean and error bars represent the standard deviation.

The analysis of fish dark–light preference revealed a clear preference for the white area independently of the treatment (F = 7.8531, *p*-perm = 0.001; F = 63.833, *p*-perm = 0.001, for 3 and 7 days after IP injection, respectively) (Figure 7). No interaction between TiO_2_ NPs and BP-3 was found for the dark–light preference.

**Figure 8:**
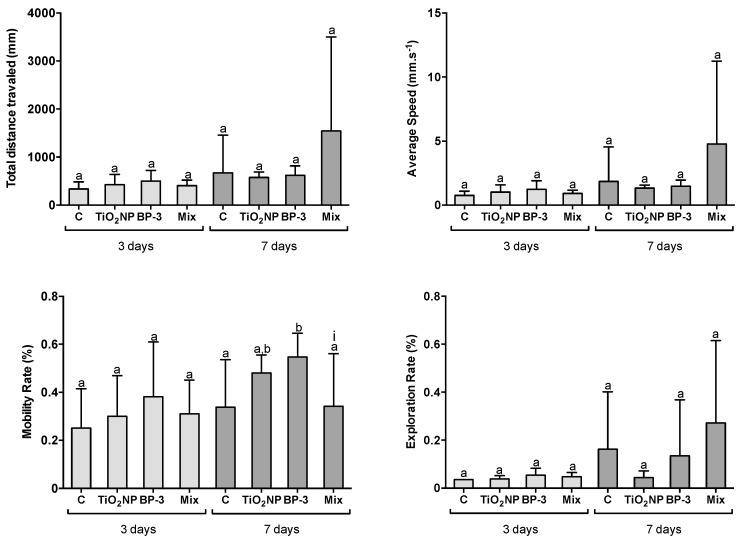
Behavioural analysis of *S. maximus*, 3 and 7 days after intraperitoneal injection of titanium dioxide nanoparticles (TiO_2_ NPs; 3.0 μg TiO_2_ NP per g fish weight), oxybenzone (BP-3; 3.0 μg BP-3 per g fish weight) or their mixture (Mix), as well as the respective control group (C). Results translate behaviour endpoints determined in the white compartment of the dark-light device (adaptation of scototaxis test). Different lower-case letters denote significant differences (*p* < 0.05). Significant interaction between TiO_2_ NPs and BP-3 is depicted by (i) (*p* < 0.05). Columns correspond to mean and error bars represent the standard deviation.

After 3 days of exposure, no alteration in the behavioural endpoints was found (F = 0.9903, *p* = 0.4122; F = 1.3228, *p* = 0.2875; F = 0.6478, *p* = 0.5911 and F = 1.5692, *p* = 0.2198 for total distance travelled, average speed, mobility rate and exploration rate, respectively) (Figure 8). After 7 days, the mobility rate was higher for BP-3 in comparison with control and the mixture (F = 3.9739, *p* = 0.0182), while the other parameters remained unchanged (F = 1.39553, *p* = 0.2656; F = 1.60369, *p* = 0.0182; F = 2.257642, *p* = 0.104 for total distance travelled, average speed and exploration rate, respectively) (Figure 8). The interaction between TiO_2_ NPs and BP-3 was significant for the mobility rate after 7 days (F = 8.749708; *p* = 0.0031).

## 3. Discussion

Sunscreens are considered contaminants of emergent concern (CEC), with little information regarding their toxicity to marine biota within environmental ranges of occurrence, as well as in mixture. Following a multi-biomarker and multi-level approach, the present work reported that inorganic and organic UV filters induced mild effects on turbot, with inconclusive information in relation to an increased impact of the mixture in comparison with the isolated chemicals. This finding reinforces the importance of increasing knowledge on UV filters mixtures, namely TiO_2_ NPs and BP-3.

### 3.1. Organ-Specific Oxidative Stress Profiles

Despite the controversy regarding the array of effects induced by NPs in fish and, specifically, TiO_2_ NPs, some of the reported observations are related with oxidative stress mediated responses [49,50,51,52,53]. On the contrary, the evaluation of BP-3 toxic mechanisms in fish barely exists. Still, oxidative stress was reported as the main cause of BP-3-induced damage in corals, marine bivalves [31,54] and in freshwater fish [55,56]. According to the present data, it can be stated that no oxidative stress occurred, since no LPO was found in any of the studied organs. However, the turbot antioxidant machinery was challenged, demonstrating different responses (either adaptive or signalling susceptibility) depending on the organ and time of exposure.

After IP injection of TiO_2_ NPs, BP-3 and its mixture, and keeping in view a temporal perspective, the intestine seemed to be the first organ targeted, followed by the liver. Moreover, the intestine showed susceptibility to the mixture, while the liver indicated adaptability to the same treatment. This interpretation relies on the observations that, after 3 days of exposure, the GPx activity in the intestine decreased in the mixture (in comparison to all the other treatments), despite the increase in GSHt content and GR activity. After 7 days, GR activity in the intestine decreased in TiO_2_ NPs (vs. all the treatments), suggesting impairment of NADPH homeostasis, since GR is an NADPH-dependent oxireductase, and the vulnerability of fish exposed to this NPs towards oxidative stress processes. NADPH is a reducing agent that is critical for an effective antioxidant response [57]. Data from the literature indicate that the fish intestine is prone to generate reactive oxygen species (ROS) due to its high content in polyunsaturated fatty acids [58]. Moreover, the fish gastrointestinal tract has been suggested to be one of the internal targets of TiO_2_ NPs, showing toxic effects related to oxidative stress responses [59,60]. Although there is no information about BP-3 effects in fish intestine, studies in neonates correlated the presence of intestinal abnormalities with the levels of BP-3 in the urine of pregnant women [61,62].

Unlike intestine, liver demonstrated to cope with the challenge induced after 3 days, depicted by the induction of SOD activity in BP-3 treatment and the increase in GSHt in the mixture. After 7 days, GR activity also increased in BP-3 and in the mixture, suggesting an adaptation mechanism to keep GSH at levels favouring a safe redox status. This adaptive ability is reinforced by the decrease in the LPO levels in these treatments. This evidence may be related with the liver role in detoxification and excretion of contaminants, namely NPs and BP-3 [22,63]. In fact, previous studies highlighted fish liver as one of the main targets of TiO_2_ NPs, both after IP injection [26] and exposure through water [64], independently of particle size (from 31 to 500 nm) [26,64]. BP-3 was also detected to a higher extent in the liver of *Mugil liza* [22] and *Gadus morhua* [65].

An overall examination of the current results suggests that kidney was not a preferential target for TiO_2_ NPs and BP-3 after IP injection, since neither the antioxidant endpoints nor LPO signalized a challenging scenario. 

The information on the distribution, metabolism and excretion of TiO_2_ NPs, in particular for fish, is still limited. However, it is believed that its excretion does not occur through the kidney, but through the liver via bile [63], which corroborates the idea that kidney is not a main target for TiO_2_ NPs. Regarding BP-3 metabolism and excretion, no information is available for fish and the majority of the data are for rats. Contrary to TiO_2_ NPs, high amounts of BP-3 and its metabolites are found in the kidney [66,67], suggesting excretion through this organ. Nevertheless, in the present study, the kidney’s role in the excretion of BP-3 cannot be confirmed. In addition, it is known that the kidney has a relevant role in excretion when exposure is via water, but not when it is via ingestion (as simulated with the IP injection), in which the enterohepatic route is privileged.

### 3.2. Hepatic Metabolic Profile

Supplementary energy supply is needed when the organisms are facing stressful circumstances, and thus diagnosis relying on both aerobic and anaerobic energetic pathways may contribute information regarding the overall energy request [68]. In this context, IDH and LDH are suitable biomarkers of the energetic metabolism, as the former participates in the aerobic and the latter in the anaerobic pathways. NADP^+^-dependent IDH is found both in mitochondria and cytoplasm catalysing the oxidative decarboxylation of isocitrate into α-ketoglutarate in the Krebs cycle, generating NADPH from NADP^+^. It is the major source of NADPH required for several important metabolic pathways, including those involved in the defence mechanisms against oxidative stress [68,69] and fatty acid metabolism [70,71]. LDH, in turn, participates in the glycolytic cycle, where it is responsible for the conversion of lactate into pyruvate, in a reversible reaction at anaerobic conditions, and is also involved in the antioxidant cell defence [72]. Fluctuations in LDH activity are often related with the increased requirements for energy during stress conditions [73].

In the present study, the alteration of these enzymatic activities was only evident after 7 days. A reduction in IDH activity was observed in the mixture, pointing out an interaction between TiO_2_ NPs and BP-3 (see further discussion in Section 3.5). Decreasing levels of IDH indicate less production of NADPH and, thus, probably less protection against oxidative stress. Simultaneously, GR activity increased in the mixture, with consequent consumption of NADPH. The source of NADPH required for the GSH conjugation pathways is likely not to provided by IDH. However, the concomitant decrease in NADPH, due to the depletion of IDH and the increase in the GR activity (which increases NADPH consumption), may suppress cells of this vital energy source, suggesting the increasing susceptibility of the liver to oxidative processes as well as to the impairment of other metabolic pathways mediated by NADPH. Since no LPO was verified in the liver, its susceptibility towards oxidative damage must be discharged. Still, it should be assumed that the depletion of IDH activity (NADP^+^-dependent) may affect fatty acid synthesis and mitochondrial function [71]. The decline in IDH was previously related with the reduction in the hepatic lipid content of catfish exposed to copper [74]. Moreover, Ziarrusta et al. [75] demonstrated that *in vivo* exposure (14 days) of gilthead seabream to BP-3-induced alterations to several pathways of lipid metabolism, also affecting liver metabolome. These findings point towards possible alterations to the fatty acid metabolism of fish exposed to mixtures of TiO_2_ NPs and BP-3. To our knowledge, there are no studies reporting NADP^+^-dependent IDH activities under the effects of TiO_2_ NPs and BP-3 in marine organisms.

LDH activity remained broadly unchanged; however, though lacking statistical significance, there seems to have been a slight increase in BP-3 treatment, suggesting the switch from aerobic to anaerobic metabolism, which can also denote an association with the higher mobility rates in this treatment (described in Section 3.4). Switching from aerobic to anaerobic metabolism has been considered a physiological adaption during exposure to xenobiotics, securing the energy supply of the cells by increasing LDH activity [76]. Increasing activities of LDH were also found in the kidney epithelial cell line (LLC-PK1) after exposure to TiO_2_ NP [77] and in the liver of fish exposed to metals [76]. However, there is no information on this metabolic shift after exposure to BP-3.

### 3.3. Neurotoxicity

The neurotoxic effects of both TiO_2_ NPs and BP-3 were recognized in mammals [78,79]; however, data in fish, in particular marine species, are still inconclusive. In the present study, no neurological disturbances of the cholinergic system were found in the brain after IP injection of TiO_2_ NPs and BP-3, isolated or in mixture, for 3 and 7 days. In the same direction, several studies with freshwater fish reported no alterations in the AChE activity in the brain after waterborne exposure to TiO_2_ NPs [46,80,81,82,83]. On the contrary, waterborne exposure of the freshwater fish *Carassius auratus* to 200 μg L^−1^ of BP-3 promoted the increase in the activity of AChE [84]. Moreover, enhancement of the activity of AChE was also demonstrated in the brain of zebrafish exposed to an effluent supplemented with TiO_2_ NPs, reinforcing that, when this NP is evaluated in mixture (natural occurring mixtures), its neurotoxicity can be promoted [83].

Interestingly, some authors found a reduction in AChE activity in fish muscle after exposure to TiO_2_ NPs while no alteration was found in the brain [80,82]. This suggests that the neurotoxic action of TiO_2_ NPs does not affect brain directly but may be related with alterations of the metabolic processes on which the nervous system is specifically dependent. This assumption is in line with the alterations found in the IDH levels after 7 days. We can also hypothesize that TiO_2_ NPs and BP-3 did not reach the brain after short-term exposure through IP injection. The permeability of the fish brain blood barrier (BBB) to TiO_2_ NP is controversial; while some authors reported accumulation of this NP in the brain after long-term dietary exposure [85], others stated that this is not completely supported and that the observed toxic effects must be the result of secondary toxicity [49,60]. On the contrary, as BP-3 is a lipophilic compound, it can cross the BBB. In fact, [86] found that this UV filter induced developmental neurotoxicity in embryos of zebrafish. Nevertheless, the same authors stated that the major neurotoxic effects of BP-3 occur in an early phase of neurogenesis. These evidences reinforce the absence of differences in the AChE levels in turbot brain.

In addition, in view of the response pattern of the intestine and liver, it is possible to anticipate a buffer effect of these organs, which prevented the distribution of BP-3 and, eventually, TiO_2_ NP, from reaching other organs, such as the brain.

### 3.4. Fish Behavioural Analysis

Behaviour comprises a set of structured and foreseeable activities aiming to maximize the fitness and survival of individuals [44]. Behavioural-related endpoints are thus valuable to address the effects of aquatic contaminants in fish by providing a whole-organism response [43,44]. In the current study, the UV-filters seem not to induce strong effects on turbot behaviour, as the only parameter that exhibited alterations was fish mobility rate, which increased after BP-3 IP injection. Compared with other fish groups, flatfishes are more sedentary and have a highly developed camouflage ability, which relies on the integration of morphological adaptations and behavioural tactics [87]. In fact, the mobility rate of turbot in all treatments was low (maximum of 0.6% during the whole survey). Despite this, it increased nearly 70% in the BP-3 treatment after 7 days, suggesting alterations on the turbot anti-predator behaviour. This increasing mobility may have induced metabolic adjustments demonstrated by the concomitant tendency towards LDH increase, suggesting the switch from aerobic to anaerobic metabolism, which might denote turbot adaptive mechanism to stress.

The effects of TiO_2_ NPs and BP-3 in fish behaviour have already been evaluated. Rainbow trout (*Onchorynchus mykiss*) exposed to waterborne TiO_2_ NPs demonstrated a decreased proportion of time spent swimming at high speed (>20 cm s^−1^) [47]. Barone et al. [24] also reported that a non-TiO_2_ NPs-based sunscreen had little impact on the swimming velocity of waterborne exposed clownfish (*Amphiprion ocellaris*). Similarly to these results, no alterations to turbot swimming behaviour were found after IP injection of TiO_2_ NPs. Regarding BP-3, the type and severity of the effects depended on the via of exposure and also on the tested concentrations. Clownfish exposed to 100 mg L^−1^ of BP-3 through water, showed atypical movements in 100% of fish during the entire assay (97 h) [24]. In addition, male Siamese fighting fish (*Betta splendens*), exposed via water to 1 mg L^−1^ of BP-3 for 28 days revealed a decrease in the maximum swimming velocity, though without alterations to the spontaneous swimming activity [88]. The exposure of the juvenile clownfish via diet to 1 μg of BP-3 per g of fish during 90 days, suggested that this UV filter might not cause a significant impairment of social behaviour [45]. In general, these findings suggested that BP-3 produced more severe effects than TiO_2_ NP- and non-TiO_2_ NP-based sunscreen, which seems to be in line with the present results.

The results for the dark–light preference indicated an almost exclusive prevalence of turbot in the light area, which, in the present case, reflects fear–avoidance behaviour or inability/less instinct to explore new environments. This is interesting, since reared flatfish tend to lack warning behaviour against predators and to adverse environmental conditions [87], which could promote more entries in the dark compartment. Another hypothesis is that, occasionally, fish could freeze during the test after choosing one of the compartments, as reported by Maximino et al. [89]. These authors pointed that this “freezing behaviour” can be related to the presence of noise and excessive movements in the experimentation room as well as due to stressful manipulations, which was not the case, as the proper conditions for behavioural evaluation were met and the choice of white compartment was recurrent.

Our data suggest low to absent effects of these UV filters on turbot behaviour after short-term exposure. This result must be endorsed by the development of new studies testing long-term exposure (e.g., re-injection of the compounds through time) and also considering different behavioural traits.

### 3.5. Identifying Chemical-Chemical Interactions under Simultaneous Exposure

Statistically significant interactions between TiO2 NPs and BP-3 were found in several of the studied parameters (GPx and GSHt in intestine; GSHt, IDH and LPO in the liver; CAT in the kidney; mobility rate), pointing out the complexity of the responses to the combination of UV filters studied in the different organs.

After 3 days of IP injection, the GPx activity in the intestine exhibited a potentiation type of interaction, as no effect was detected following the isolate exposure to TiO2 NPs or BP-3, but an inhibitory effect was potentiated in relation to this enzymatic antioxidant when fish were exposed to the mixture. Concomitantly, GSHt exhibited an infra-additive interaction, since the mixture exhibited the same adaptive response as the isolated UV filters. In both cases, the previously mentioned interactions (day 3) appear to contribute to increased risk, limiting the enteric antioxidant capacity. Differently, GR activity seems to have been exempt from this pressure, since it demonstrated an additive effect.

A significant interaction between TiO_2_ NPs and BP-3 was found for CAT activity in the kidney (3 days). This interaction is not easily interpreted, but it can be suggested that a slight tendency (statistically non-significant) of TiO_2_ NPs to restrain CAT activity was potentiated by BP-3 in the mixture. Anyhow, a minor toxicological meaning should be attributed to this process, since, as previously pointed out, kidney was not a primary target of these compounds via IP injection.

In the liver, the content of GSHt in fish exposed (3 days) to the mixture also reflected a potentiation type of interaction of these UV filters, but, in this case, it seems to translate to an improved adaptive capacity to cope with the combined exposure. Concurrently, the significant interaction found for LPO, although not fully supported by statistics (only a slight and statistically non-significant tendency to increase was found), may translate to a subjacent antagonism, perceptible in the mixture, preventing lipidic damage. A causal relationship can be identified between the interferences described for the liver on day 3, highlighting GSHt as the main contributor to avoid oxidative stress in this key organ as well as a favourable outcome of the UV filter interaction.

Keeping the focus on the liver, after 7 days of IP injection, IDH activity demonstrated a potentiation effect, revealing a strong inhibitory effect of this enzyme in fish exposed to the mixture and, thus, a decrease in the hepatic aerobic metabolism. Concomitantly, the significant interaction on the mobility rate points out an antagonism between the two UV filters, as the co-exposure to TiO_2_ NPs prevented the excitatory effect of BP-3. Keeping in mind a mechanistic interpretation, it can be hypothesised that, in fish exposed to the mixture, potential to alter anti-predator behaviour was masked by the impossibility of carrying out the required metabolic adjustments.

In some circumstances, despite that no statistically significant interactions were signalized, the comparative analysis of the results profile for isolated and mix exposures suggests some further chemical–chemical interactions as well as additivity phenomena. Hence, after 7 days, an antagonism seems to have occurred between the two UV filters concerning GR activity in the intestine, resulting from the interference of BP-3 in the effect induced by TiO_2_ NPs (enzymatic inhibition). Denunciating a complex pattern of interference, in the precedent sampling moment (3 days) the GR activity in intestine reflected additivity towards its induction.

In liver, after 7 days, a potentiation was found in GR activity, as TiO_2_ NPs, showing no effect, were, however, able to accentuate the effect of BP-3 when exposed in combination. This supra-additive type of interaction, in this particular context, seems to strengthen the hepatic redox buffering, which is translated into a potentially more favourable GSH/GSSG ratio.

Globally, our findings demonstrated that the type of interaction was dependent on the organ and post-injection time. Hence, on day 3, the majority of the interactions were found in the intestine and apparently unfavourable, pointing towards increasing turbot susceptibility. In contrast, in the liver, the interactions identified at the same time suggested a favourable action of the UV filters in mixture, demonstrating adaptive mechanisms. Reinforcing the complexity of interaction profiles and the preponderance of the factors organ and time, at day 7 post-exposure, the intestine signalized a favourable effect of the UV filters in mixture, while liver highlighted both unfavourable and favourable interferences. The ecological risk, given by the behavioural endpoints, also signalized an interaction between the UV filters. However, it was not possible to classify this as favourable or unfavourable since, in the mixture, the excitatory behaviour of BP-3 was probably masked by the metabolic inability to take over the necessary adaptations, as the inhibition of the aerobic metabolism (IDH depletion) was not supported by the switch to the anaerobic metabolism (no alteration of LDH).

This evidence are a clue about the distribution pathways of these chemicals after IP injection, highlighting the intestine as the first target organ, followed by the liver. This also reinforces the role of these organs in the uptake and distribution of xenobiotics via ingestion, in particular the central role of the liver in the detoxification processes. Moreover, the different sensitivity/responsiveness of each organ suggests a toxicodynamic interaction of these UV filters as they influence the type of biochemical responses of each organ.

Soler de la Vega et al. [90] also found an interaction mechanism between TiO_2_ NPs and BP-3 in a *Daphia magna* assay. Contrary to our findings, in which most of the interactions mitigated the isolated effect of these compounds, these authors found a synergistic Trojan horse-like action between these UV filters, since their mixture showed greater effects than those estimated for additivity, based on the toxicity of isolated UV filters. They pointed that the increasing toxicity of the mixture is mainly related to two factors: BP-3 conversion into a more toxic metabolite, induced by ROS produced by TiO_2_ NPs, and the increasing bioavailability of BP-3 due to the presence of large aggregates of TiO_2_ NPs, which offers a higher contact surface for BP-3, thus enhancing its internalization. As TiO_2_ NP contributes to the increasing bioavailability of BP-3 by altering its uptake and distribution, the underlying interaction found in the work of Soler de la Vega et al. [90] seems to rely on a toxicokinetic mechanism. Due to the adopted approach, this cannot be confirmed by the present work, but we hypothesized a toxicodynamic mechanism of interaction between these UV filters. This evidence denotes that the interaction mechanisms of the same mixture of compounds might be dependent on the organism, pathway and time of exposure, as well as on the tested concentrations.

## 4. Materials and Methods

### 4.1. Fish Maintenance

Turbot (*Scophthalmus maximus*) juvenile specimens (total weight 19.6 ± 3.37 g; total length 10.2 ± 0.53 cm) were obtained from a local fish farm (Acuinova, Portugal). At the laboratory, fish were acclimatized for 4 weeks in a closed-system recirculation arrangement of polyvinyl tanks containing 50 L of artificial seawater (ASW). Water parameters were regularly controlled with average values: salinity = 34 ± 2.1, temperature = 18.3 ± 0.4 °C, pH = 8.05 ± 0.23, unionized ammonia (NH_3_) 0.005 ± 0.11 9 mg L^−1^ and dissolved oxygen 7.9 ± 0.9 mg L^−1^. Fish were fed daily with species-specific commercial pellets provided by Acuinova. A weekly 25% water change was performed to maintain constancy of the parameters.

This study was conducted in accordance with the European Union guidelines concerning the protection and animal welfare (Directive 2010/63/EU) and authorized by the Commission Responsible for Experimentation and Animal Well-being of the Department of Biology of the University of Aveiro (CREBEA-03/2018, 14-12-2018).

### 4.2. Preparation and Characterization of Titanium Dioxide Suspensions

Titanium dioxide nanopowder (TiO_2_ NPs), namely Aeroxide^®^P25 (declared purity ≥ 99.5% CAS# 13463-67-7), was supplied by Merck (Sintra, Portugal). Crystalline phase and crystallite size were identified by the X-ray diffraction technique-XRD using a Philips X’Pert MPD X-ray powder diffractometer (Philips, Netherlands, Almelo) equipped with a Cu Kα monochromatic radiation source (λαK = 1.54060 Angstron). TiO_2_ NPs stock suspension (15 mg mL^−1^) was prepared in distilled water by sonication with an ultrasonic processor (Sonics vibra cell, Newtown, USA), for 20 min at 100W, with 5:1 pulses on/off. The dispersion was performed in an ice bath. The working suspension (0.3 mg mL^−1^) was prepared in saline solution (NaCl; 9 g L^−1^) in a ratio of 0.1:49.9 *v/v* of DMSO and TiO_2_ NPs in saline solution. DMSO was added to the working suspension to eliminate the possibility of vehicle interferences when testing the mixture, since BP-3 solution was prepared in Dimethyl sulfoxide (DMSO; CAS# 67-68-5, Merck, Sintra, Portugal) (see Section 4.4).

The TiO_2_ NPs structure (stock and working suspensions) was confirmed by scanning transmission electron microscopy (STEM), using a JEOL 2200FS, JEOL Ltd., Japan model.

### 4.3. Preparation of Oxybenzone Solutions

Oxybenzone (BP-3), namely 4-methoxy-2-hydroxybenzophenone (declared purity = 99% and CAS# 131-57-7) was supplied by Merck (Sintra, Portugal). Due to the low solubility of BP-3 in water, stock solution (150 mg mL^−1^) was prepared in DMSO. BP-3 working solution (0.3 mg mL^−1^) was prepared in a ratio 0.1:49.9 *v/v* of BP-3 in DMSO and saline solution (NaCl; 9 g L^−1^).

### 4.4. Exposures to TiO_2_ Nanoparticles and Oxybenzone

The experimental assay aimed to evaluate the multi-level effects of exposure to 3.0 μg (TiO_2_ NPs and/or BP-3) per g of fish weight (mean volume of 200 μL per fish of 20 g, containing 60 μg of contaminant), administered by intraperitoneal (IP) injection. IP injection is the method most similar to oral administration [91], mimicking the entry of the tested compounds into the organism via the food chain and/or via contact with the sediments. This is particularly relevant for flatfishes, since they are ground-dwelling and burying animals. Moreover, IP injection is suitable for small specimens and were shown to be effective for studies with TiO_2_ NPs in marine fish [26], avoiding NPs’ aggregation and agglomeration in aqueous saline solutions.

The experiment included four treatments: control (C, fish injected with 0.1:49.9 *v/v* of DMSO and saline solution), TiO_2_ NPs treatment (fish injected with TiO_2_ NPs suspension), BP-3 treatment (fish injected with BP-3 solution) and mix treatment (fish injected with a solution corresponding to the mixture of TiO_2_ NPs and BP-3 (3.0 μg TiO_2_ NPs and 3.0 μg BP-3 per g of fish weight).

All fish were injected with the respective working solution prepared in 0.1:49.9 *v/v* of DMSO and saline solution. The same low percentage of DMSO (0.02 g kg^−1^ fish weight) was used in all treatments. This concentration was within the range of non-detectable toxicity [92].

Fish were sampled 3 and 7 days post-injection. Each experimental group/treatment consisted of three 5-L aquariums, each containing five fish (15 fish per condition—1 L of water per fish). In total, there were 24 aquariums (12 per exposure duration), all with identical water conditions: dissolved oxygen = 7.9 ± 0.6 mg L^−1^, pH = 8.15 ± 0.01, NH_3_ = 0 to 0.25 mg L^−1^, temperature = 17.3 ± 0.6 °C and salinity = 35 ± 0.0.

### 4.5. Sampling and Biochemical Analyses

At the end of each exposure period (i.e., 3 and 7 days), fish were sacrificed, properly bled, and liver, kidney, brain and intestine were sampled and immediately frozen in liquid nitrogen followed by storage at −80 °C. Each organ sample was homogenized in a chilled phosphate buffer (0.1 M, pH 7.4) using a Potter–Elvehjem homogenizer (Glas-Col, Terre Haute, USA). A 1:6 dilution (organ (g): buffer volume (mL)) was used for all organs except brain (where 1:10 dilution was used). The homogenate was then divided in two aliquots: for lipid peroxidation (LPO) and to obtain the post mitochondrial supernatant (PMS). For LPO, 100 μL of homogenate was collected with 10 μL of butylatedhydroxytoluene 4% and frozen immediately on liquid nitrogen. PMS was prepared by centrifugation in a refrigerated centrifuge (Eppendorf 5415R) at 4 °C for 20 min at 12,000 rpm. Aliquots of PMS were collected, frozen in liquid nitrogen and stored at −80 °C until analyses. An aliquot for total glutathione (GSHt) quantification was also stored. PMS for GSHt was prepared by precipitating the non-soluble PMS protein in 12% TCA (1:2 dilution). Briefly, PMS tubes were incubated at 4 °C for 60 min, then centrifuged at 12,000× *g* for 5 min at 4 °C. The supernatant was collected, frozen in liquid nitrogen and stored at −80 °C until GSHt analysis. All reagents used for biochemical analyses were supplied by Merck (Sintra, Portugal).

LPO test was based on Bird and Drapper [93] and adapted by Filho et al. [94] with some modifications. The presence of thiobarbituric acid reactive substances (TBARs) was measured on a microplate at 535 nm. The malondialdehyde (MDA) concentration, i.e., TBARS equivalent, was calculated using an extinction coefficient of 1.56 × 10^−5^ M^−1^ cm^−1^ and expressed as nmol of TBARS formed/mg of protein.

Catalase (CAT) activity was performed according to Claiborne [95] and Giri et al. [96]. The absorbance was read at 240 nm, during 3 min at 10 s intervals at 25 °C. The CAT activity was expressed in μmol H_2_O_2_ consumed/min/mg protein (Ԑ = 43.5 M^−1^ cm^−1^).

Total glutathione quantification (GSHt) was done according to the methodology of Baker et al. (1990) [97] adapted by Vandeputte et al. [98] for microplate. When GSH is oxidized to GSSG it gives 5-thio-2-nitrobenzoic acid (TNB). This formation can be quantified by spectrophotometry and is proportional to the sum of GSH and GSSG. The absorbance was read at 415 nm for 7 min at 30 s intervals at 25 °C. The formation of TNB concentration was expressed as nmol TNB conjugated/min/mg protein (Ԑ = 14.1 × 10^3^ M^−1^ cm^−1^).

The activity of glutathione reductase (GR) was directly determined by the oxidation of NADPH according to Cribb et al. [99]. The absorbance was read at 340 nm for 5 min with 30 s intervals at 25 °C and expressed as nmol NADPH oxidized/min/mg protein using a Ԑ = 6.22 × 10^3^ M^−1^ cm^−1^.

The activity of glutathione peroxidase (GPx) was quantified by the oxidation of NADPH to NADP^+^ at 340 nm, according to the method described by Mohandas et al. [100], modified by Athar and Iqbal [101]. The absorbance was monitored for 5 min at intervals of 30 s at 25 °C and expressed as nmol NADP^+^/min/mg protein (Ԑ = 6.22 × 10^3^ M^−1^ cm^−1^).

To determine the activity of superoxide dismutase (SOD) a spectrophotometric enzymatic kit (RANSOD TM, Randox) was used and adapted to a microplate. Xanthine and xanthine oxidase were used to generate superoxide radicals, which react with 2-(4-iodophenyl)-3-(4-nitrophenol)-5-phenyltetrazolium chloride (INT) originating from a red dye of formazan. The absorbance was read at 550 nm during 3.5 min with 30 s intervals at 25 °C. SOD activity was expressed as SOD/min/mg protein, which, in a unit of SOD, is what causes the inhibition of 50% of the INT reduction rate.

Isocitrate dehydrogenase (IDH) activity was determined in the liver according to Ellis and Goldberg [102], adapted to microplate [103]. The absorbance was read at 340 nm at 25 °C for 2.5 min at 10 s intervals and the results were expressed as nmol NADPH-regenerated/min/mg protein (Ԑ = 6.22 × 10^3^ M^−1^ cm^−1^).

Lactate dehydrogenase (LDH) activity was determined in the liver according to Vassault [104], followed by Diamantino et al. [105] adaption to microplate. The absorbance was read at 340 nm at 25 °C for 2.5 min at 10 s intervals and the results were expressed as nmol NAD^+^/min/mg protein.

Acetylcholinesterase (AChE) activity was determined in the brain according to Ellman et al. [106], were thiocholine, a product of the reaction, bind with 5,5′-dithiobis-(2-nitrobenzoic acid) (DTNB) forming a yellow compound. The absorbance was read at 412 nm at 25 °C for 15 min at 5 min intervals and calculated as nmol substrate hydrolysed/min/mg protein (Ԑ = 1.36 × 10^4^ M^−1^ cm^−1^).

The total protein content was determined in the PMS homogenates and in LPO aliquots according to the Biuret method [107], using bovine serum albumin as a standard. Absorbance reading was done at 550 nm.

All endpoints were measured using a SpectraMax 190 microplate reader (Molecular Devices LLC, California, United States).

### 4.6. Fish Behavioural Assessment

**Figure 9:**
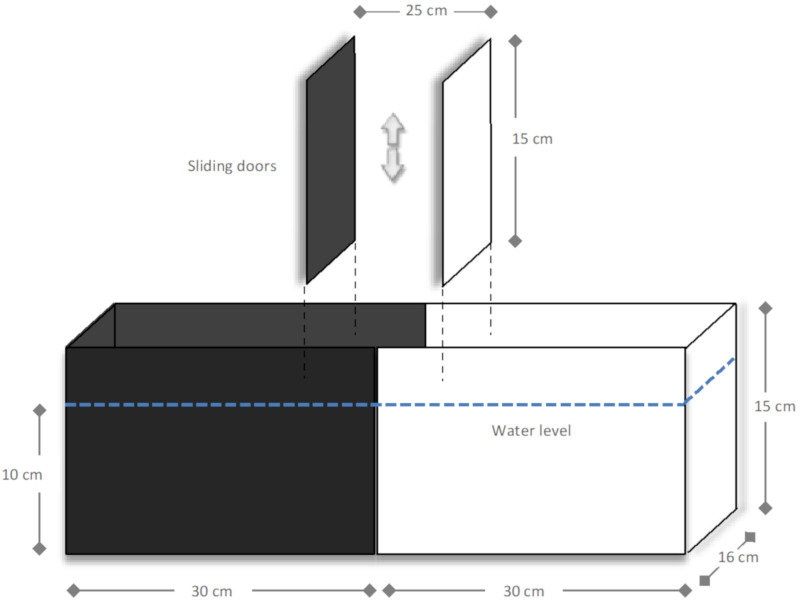
Schematic overview of the dark–light device used for fish behavioural analysis protocol, as adapted from [89]. The apparatus consisted in a non-reflective aquarium made of opaque acrylic of 60 length × 16 width × 15 cm height, with a central compartment of 25 × 16 × 15 cm, and 10 cm water column.

Turbot preference for dark or light (scototaxis test) was assessed in an appropriate device, as proposed by Maximino et al. [89,108] (Figure 9). The behavioural scototaxis test in fish (cyprinid and non-cyprinid species) was developed to address its preference for a light or dark area, considering activity-exploration and fear-avoidance related responses. This test aims producing a well-defined conflict condition for fish, contributing with information on fish natural trend to explore when dealing with hostile situations [89]. Fish preference for an area is based on their crypsis process which, in the majority of the species, relies on a preference for the dark area (scototaxis) [89]. Flatfish have an outstanding ability to adapt to their background by shifting their body pattern [87]; in addition, flatfish cryptic colouration may be altered in animals reared in light-coloured tanks and without sediments [109], as in the present study, with fish showing a blanched appearance in the ocular side. Hence, the acclimation for 4 weeks in white tanks conceivably introduced a preference for the white area (contrary to the scototaxis assumptions for most species). In agreement with this, the presence of turbot in the dark/black area (as perceived by duration and/or entries) should reflect an innate motivation to explore new environments, whereas increased activity in the light/white compartment may indicate a preference for a protected/shelter area [89].

The apparatus consisted in a device made of opaque acrylic (60 length × 16 width × 15 cm height), as shown in Figure 9, divided equally into one-half black and one-half white. The device contained sliding central doors, coloured with the same colour of the respective aquarium side (black or white), defining a central compartment of 25 × 16 × 15 cm. To avoid the fish tendency towards shoaling behaviour in relation to their own reflection, the material chosen for the device is non-reflective. The device was filled with seawater at 10 cm height, corresponding to 9.6 L total volume. The procedure for fish behavioural assessment using the dark–light device followed the descriptions of Maximino et al. [108]. Briefly, fish (*n* = 9 for each treatment) were transferred individually from the experimental tanks to the device and placed in the central compartment for 5 min for acclimation to the new environment. After this acclimation time, the sliding doors were removed and the animal was left to freely explore the whole area of the apparatus. Fish behaviour was recorded (video-taped with a digital camera aligned with the centre of the device) during the test duration (5 min) for posterior analysis. The preference for the dark or light area was assessed by recording the duration, in seconds, of the entrances in each area.

For further analyses of behavioural endpoints, the ToxTrac software, version 2.84, was used (software freely available at sourceforge.net/projects/toxtrac) [110,111]. This software allows for the evaluation of parameters with ecological relevance such as locomotor activity and the time spent exploring [110]. ToxTrac will detect and track animals in rectangular sections of the image containing the arenas (manually defined) where the user wishes to perform the observations. The algorithm to detect the animals in the tracking areas is defined by pre-processing the image to avoiding distortion, separate the tracking areas (segmentation) and discarding objects that exceed the maximum and minimum sizes (filtering). The obtained coordinates are in pixel and relative to the individual after removing distortion. Then, a translation is made and coordinates are finally expressed in millimetres and time in seconds. A fixed value for minimum and maximum size in pixels of the detected bodies is defined, as well as a fixed threshold for minimum and maximum intensity level (1–255) for the detected animal. Then, adopting an assay time of 5 min, the following behavioural parameters were obtained:

- Total distance travelled (mm)—Represents the sum of all the displacements for each fish while it moved freely during the whole time period of the test; only movements that were able to move the centre of mass were considered displacement;
- Average speed (mm s^−1^)—Represents the average of instantaneous speeds for each fish during the whole assay time, where the instantaneous speed S_i_ corresponding to the time t_i_ is calculated according to the formula
  (1) $$s_{i}=\frac{\sqrt{{\left(x_{i+c}+x_{i-c}\right)}^{2}+{\left(y_{i+c}-y_{i-c}\right)}^{2}}}{t_{i+c}-t_{i-c}}$$
  c is a sampling distance.
- Mobility rate (%)—The percentage of whole assay time that each fish remained in motion for, including movements in great lengths or within the same area;
- Exploration rate (%)—Each recorded arena was divided into regular non-overlapping zones in a total of 28 zones; then, the use of each zone was computed for each arena by dividing the total number of detections in the zone by the total number of detections. This parameter can provide information on to what extent the exploratory behaviour of fish was affected.

Due to the cryptic ability of flatfish and their matching with the background, both the software and visual registration could not be performed in the dark area. Thus, the described parameters were uniquely analysed in the light area of the device.

### 4.7. Statistical Analysis

The effects of the different treatments (independent variable) on the biochemical and behavioural endpoints (dependent variables) were analysed per exposure moment using one-way ANOVA. The effect of the mixture treatment was tested with a two-way ANOVA, based on the presence of either TiO_2_ NPs or/and BP-3. Whenever the interaction between the presence of TiO_2_ NPs and BP-3 was significant, an effect of the mixture was assumed. Graphical validation tools were used to verify ANOVA assumptions. Whenever these were not fulfilled, data were analysed with generalized least squares (GLS) statistical mixed modelling that allows for unequal variance [112]. The results were expressed as estimated mean and standard error. All the statistical tests were considered significant when *p* < 0.05.

The effects of the different treatments on the preference for dark–light preference was analysed with permutational analysis of variance (PERMANOVA), using PRIMER v6 and PERMANOVA + v1 software (PRIMER-E, Ltd., Auckland, New Zealand), based on Euclidean distance matrices with unrestricted permutations. Area and treatment were treated as crossed categorical factors.

The statistical analyses were performed using the ‘R’ statistical and programming environment [113]. GLS models were implemented using the ‘nlme’ package (linear and nonlinear mixed effects models; [114]) and PERMANOVA analyses using the ‘vegan’ package (Community Ecology Package; [115]). 

## 5. Conclusions

The present study found that TiO_2_ NP and BP-3, isolated and in mixture, have a mild impact under the tested experimental scenario, exhibiting no unequivocal signals of toxicity after IP injection, as no oxidative stress (absence of LPO in all the addressed organs) and neurotoxicity (absence of alterations in the AChE activity in the brain) were found. The intestine and liver were preferentially targeted, while kidney and brain were unaffected. These UV filters also induced little (BP-3) or no behavioural alterations. Nevertheless, their presence in the mixture impaired fish bioenergetics by inhibiting the aerobic hepatic metabolism. Both infra- and supra-additive interactions between TiO_2_ NP and BP-3 were perceived, with a toxicodynamic nature, resulting in either favourable or unfavourable toxicological outcomes, which were markedly dependent on the organ, parameter and post-injection time. The combined exposure to the UV filters did not show a consistent increment of toxicity in comparison with the isolated exposures, which is an ecologically relevant finding and provides key information regarding the formulation of environmentally safe sunscreen products.

## Data Availability

The data presented in this study are available on request from the corresponding author. The data are not publicly available since there are still several outputs to be further evaluated under different perspectives.
